# The Involvement of Prolactin in Stress-Related Disorders

**DOI:** 10.3390/ijerph20043257

**Published:** 2023-02-13

**Authors:** Agata Faron-Górecka, Katarzyna Latocha, Paulina Pabian, Magdalena Kolasa, Iwona Sobczyk-Krupiarz, Marta Dziedzicka-Wasylewska

**Affiliations:** 1 Department of Pharmacology, Maj Institute of Pharmacology, Polish Academy of Sciences, 31-343 Kraków, Poland; 2 Department of Infectious and Tropical Diseases, Jagiellonian University Medical College, 30-688 Kraków, Poland

**Keywords:** prolactin, stress, neuropsychiatric disorders, hyperprolactinemia

## Abstract

The most important and widely studied role of prolactin (PRL) is its modulation of stress responses during pregnancy and lactation. PRL acts as a neuropeptide to support physiological reproductive responses. The effects of PRL on the nervous system contribute to a wide range of changes in the female brain during pregnancy and the inhibition of the hypothalamic–pituitary axis. All these changes contribute to the behavioral and physiological adaptations of a young mother to enable reproductive success. PRL-driven brain adaptations are also crucial for regulating maternal emotionality and well-being. Hyperprolactinemia (elevated PRL levels) is a natural and beneficial phenomenon during pregnancy and lactation. However, in other situations, it is often associated with serious endocrine disorders, such as ovulation suppression, which results in a lack of offspring. This introductory example shows how complex this hormone is. In this review, we focus on the different roles of PRL in the body and emphasize the results obtained from animal models of neuropsychiatric disorders.

## 1. Introduction

Prolactin (PRL) has more than three hundred different biological functions and is well-recognized for its maternal functions. Even the name of this hormone derives from its ability to stimulate lactation in response to a baby’s suckling stimulus. However, the role of this hormone in stress responses is not without significance, which is the reason it is often called the stress hormone. Stress is a contributor to many disorders, especially neuropsychiatric disorders; therefore, this review will primarily focus on the role of PRL as a potential target in stress-related disorders and the consequences that arise when PRL dysregulation is the cause of such disorders.

## 2. Prolactin and Its Receptors

PRL is a polypeptide encoded by a single gene located on the short arm of the sixth chromosome in humans [1]. PRL consists of 199 amino acids in humans, whereas in rats and mice, which are the animals most used in research, pituitary PRL consists of 197 amino acids, with a molecular weight of 23 kDa [2]. While the 23 kDa of PRL is the main variant, other biologically active forms have also been characterized [3]. Chromatographic analyses of blood serum and pituitary extracts have revealed four forms of PRL [4,5]: low prolactin (little PRL)—M.W. 22–25 kDa; high prolactin (big PRL)—M.W. 40–50 kDa; very large, aggregated prolactin (big-big PRL)—M.W. > 50 kDa; or macro-PRL, which has very low biological activity and appears to predominantly circulate in postmenopausal women. The main site of PRL synthesis and secretion is the anterior pituitary gland, but the uterus, mammary glands, prostate, skin, fat cells, or immune cells are also such sites. The tuberoinfundibular dopaminergic pathway (TIDA) is involved in the inhibitory control of PRL secretion [6]. The dopaminergic neurons that control PRL secretion are in the arcuate nucleus of the hypothalamus. When dopamine is secreted into the pituitary portal vasculature, it acts on dopamine D2 receptors (D2R) that are present on the surface of lactotroph cells, thereby inhibiting PRL release. PRL, in turn, participates in negative feedback to control its release by increasing the activity of tyrosine hydroxylase and, thus, the synthesis of dopamine in TIDA neurons. PRL functions are mediated by PRL receptors (PRLRs) which belong to class 1 of the cytokine receptors superfamily [7]. PRLRs comprise multiple isoforms, including long, intermediate, and short isoforms (PRLR-L, PRLR-I, and PRLR-S, respectively) that are produced by alternative splicing from a single gene [6]. PRLR expression has been detected in different regions of the brain, with the highest level in the choroid plexus (ChP) [8]. PRL likely crosses the blood–brain barrier through a receptor-mediated mechanism that occurs in the cells of the ChP [9]. However, studies using PRLR knockout mice have shown that PRL transport does not depend on the PRLR; rather, it depends on another mechanism which has not been identified yet [10]. Recent evidence suggests that the ChP is an alternative source of PRL for the brain that might impact the neurogenesis of olfactory neurons in the subventricular zone (SVZ) given its proximity to the ChP [11].

## 3. PRL and Stress

The neurochemical marker of stress is an increase in the activity of the hypothalamus–pituitary–adrenal (HPA) axis. The HPA axis plays an important role in the processes of both adaptation and the body’s response to stress. HPA coordinates the secretion of glucocorticoids (such as cortisol) from the adrenal cortex into the blood; therefore, it is responsible for preparing the body for the fight or flight response. A series of hormonal changes that occur due to stress factors along the HPA axis are initiated by the neurohormone corticotropin-releasing hormone (CRH). CRH stimulates the production and secretion of adrenocorticotropic hormone (ACTH) in the pituitary gland, which is synthesized by cells of the anterior lobe of the pituitary gland. ACTH via the bloodstream enters cells of the adrenal cortex and stimulates them to produce corticosteroids—more specifically, glucocorticoids, which include cortisol. The mechanism by which PRL affects the stress response is not currently known but may involve effects on corticotropin-releasing hormone (CRH) neurons in the medial hypothalamic nucleus (PVN). These neurons integrate signals from stress-processing circuits to control the pituitary and adrenal activation of the hypothalamic–pituitary–adrenal (HPA) axis. Chronic intracerebral administration of PRL significantly reduces the stress-induced activation of the HPA axis in virgin rats, while the opposite effect is seen with the downregulation of central PRLRs [12,13]. During lactation, which is a characteristically hyperprolactinemic state, the responsiveness of the HPA axis to various stress factors is suppressed, and this effect is reversed when central PRLRs are blocked [14]. These results indicate that the chronic elevation of PRL signaling in lactation may play an important role in the regulation of HPA axis reactivity during this time.

Although the secretion of PRL is influenced by many environmental factors (i.e., circadian rhythms or seasonal changes), PRL also belongs to a group of hormones that is strongly regulated by stress. Results obtained in animal models have indicated that the secretion of this hormone is dependent on the type and intensity of the stress. Immobilization stress had a biphasic effect on serum PRL levels—an early, short phase of stimulation was followed by a long phase of inhibition in male rats [15]. In studies using the chronic mild stress (CMS) model, which is a well-validated animal model of depression, the serum PRL levels of stressed animals measured after two weeks of stress did not change compared with those of unstressed animals, while after seven weeks of stress, a decrease in PRL levels measured in the serum of the rats was observed [8]. Similar effects were observed using this model in mice of both sexes, although the effect was stronger in female mice undergoing stress [16]. Thus, it appears that exposure to the same stressor causes adaptation to the stimulus, resulting in a lower physiological response. Therefore, acute, unpredictable stress causes an increase in serum PRL levels [17], while repeated stressors do not increase PRL levels. In addition, regulation at the level of PRL seems to be crucial. Different stressors induce PRL release from the pituitary [18], and circulating PRL enters the central nervous system (CNS) through the ChP [11]. In a study that used CMS, an increase in PRLR in the ChP was observed after two weeks of stress, while prolonged stress did not affect PRLR levels in this structure [8]. It has been shown that the autoregulation of PRL is possible through fluctuations in the concentration of this hormone in cerebrospinal fluid (CSF) [19]. Besides its barrier function between the periphery and the CSF, the ChP has an important role in CSF production and the synthesis and secretion of proteins and other signaling molecules that impact the development and functions of the brain, including neurogenesis. In addition, the peripheral and central administration of PRL has been shown to increase PRLR expression in the ChP [20,21], confirming the role of this structure in PRL regulation.

Some early research showed that childhood traumatic experiences (e.g., exposure to an absent, alcoholic, or violent father) predisposed women to develop hyperprolactinemia later in life [22]. However, in more recent studies involving patients with post-traumatic stress disorders (PTSD) (mainly war veterans), plasma PRL levels decreased [23,24], did not change [25,26], or increased [27,28]. In other studies, war veterans with or without PTSD showed significantly enhanced PRL suppression in response to dexamethasone compared with healthy control participants [29], which may imply that the PRL response to dexamethasone reflected combat exposure rather than PTSD. It was also shown that PTSD patients demonstrated a decreased PRL response to the serotonin-releasing agent (d-fenfluramine) compared with healthy controls [27]. Analyses of the plasma concentrations of lipids and several stress hormones in patients with PTSD revealed a sex-dependent association of the lipoprotein profile and level of stress hormones, including PRL. Specifically, the effect of PRL on LDL was affected by both sexes and the presence of PTSD. Generally, women showed an inverse correlation between PRL and LDL (i.e., decreased LDL levels were associated with increases in prolactin levels). However, male PTSD patients show decreased LDL and increased prolactin, while male controls show increased LDL and increased PRL [30]. These results may indicate that prolactin regulation might be disrupted in cases of PTSD compared with controls, although there is still a lack of clear evidence about this phenomenon.

## 4. PRL and Neuropsychiatric Disorders

Among the environmental factors that influence the formation of depression, the most important factor is stress. More than half of patients with depression show signs of excessive activation of the HPA axis and a disturbed negative feedback loop. Moreover, effective therapy with antidepressants leads to the normalization of this hyperactivity. In contrast, antidepressants directly inhibit the activity of the HPA axis.

**Figure 1:**
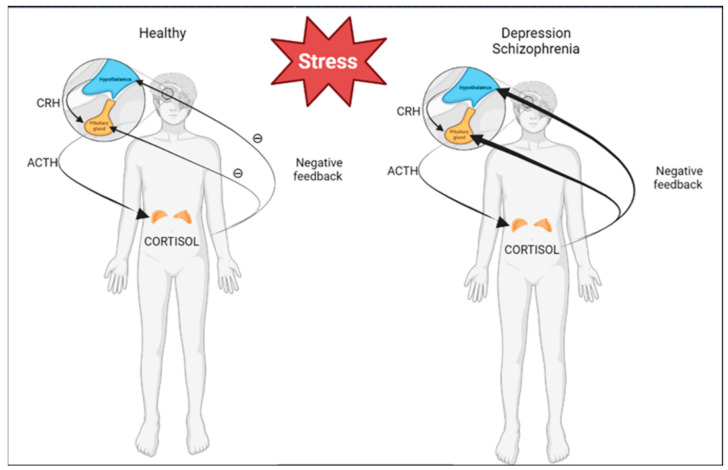
Differences in HPA axis activities of healthy patients and patients with depression or schizophrenia. This figure was created with BioRender.com.

Differentiated activity of the HPA axis was found in patients with schizophrenia, including a higher rate of suppression of the dexamethasone trial and higher levels of salivary cortisol, which were risk factors for psychosis in adolescents at risk of developing schizophrenia [31,32] (Figure 1).

Since stress is an important contributor to the etiopathogenesis of disorders in most neuropsychiatric diseases, the stress hormone PRL may have important diagnostic significance. In addition, it should be noted that dopamine receptors are an important regulator of PRL secretion, which is an important component of many mood-related disorders. Both depression and schizophrenia closely depend on levels of secreted dopamine. The dopaminergic hypothesis of schizophrenia was created based on the mechanism of the action of antipsychotic drugs and amphetamine-induced psychotic symptoms, and it has not been disproved, despite other discoveries. Additionally, one of the two core symptoms of depression is anhedonia, the substrate of which is mesolimbic dopamine. Anhedonia is also included in negative symptoms of schizophrenia; therefore, both depression and schizophrenia closely depend on levels of secreted dopamine [33]. In the case of depression, the disruption of its secretion leads to an impaired motivation to act, or apathy, while the observed excess dopamine in schizophrenia causes side effects in the form of delusions. The main mechanism of action of antipsychotic drugs focuses on blocking dopamine receptors that are widely distributed in the brain, which leads to side effects such as hyperprolactinemia. The involvement of PRL in these disorders is very complex and is not necessarily merely an effect resulting from complex processes in the diseased brain—it is also the cause of many other disorders. PRL may provide a “window into the brain” through which we can observe the effects and causes of neuropsychiatric disorders.

### 4.1. Depression

As mentioned above, long-term stress is believed to be a major contributing factor to depression. Recent published results indicated that in untreated women with MDD, the plasma levels of PRL were significantly higher compared with those of healthy controls, as were those of women compared with men. Moreover, the level of PRL was significantly correlated with heart rate and a wide range of self-reported measures of stress and psychopathology, including anxiety, hostility, and somatization [34].

In our study, which used the animal model of depression and the chronic mild stress paradigm (CMS), we treated rats with imipramine (IMI), which is an antidepressant drug that is widely used in preclinical studies. In our previous publications, we showed a correlation between high PRL levels in rats and therapeutic responses in the CMS model. Animals with high basal levels of PRL responded poorly to IMI treatment. This drug inhibits not only the reuptake of serotonin, but also norepinephrine and dopamine; therefore, it is reasonable to suppose that the dopaminergic system may also play a significant role in these phenomena. The observed negative correlation between baseline PRL levels and responses to drug administration in the CMS model suggests that PRL may predict responses to the pharmacotherapy of depression [35]. This hypothesis is confirmed by the observation that in patients with major depression, after electroconvulsive therapy, pharmacotherapy, and psychotherapy, high PRF (prolactin secretion responses to fenfluramine) was positively correlated with responses to treatment [36]. The results are also consistent with studies by Porter et al. [37], who showed a significant negative correlation between clinical responses and baseline PRL levels in a group of people with high tryptophan levels. It seems that an increased sensitivity of 5HT1A receptors to serotonin in response to a decreased tryptophan availability may be reflected in an increased baseline level of PRL. The influence of antidepressant treatment on PRL levels is less known [38,39]. A study by Reeves et al. in 2016 did not show a relationship between the use of antidepressants and increased PRL concentrations; however, they reported increases in PRL concentrations after antidepressant treatment in obese women [40]. This supports the equally important aspect that depression can be treated not only as a primary phenomenon, but also as a secondary phenomenon resulting from other disorders. As symptoms such as a depressed mood, anxiety, irritability, low stress tolerance, and full depressive syndrome are often reported in patients with hyperprolactinaemia, and the normalization of PRL and treatment with bromocriptine reduces depressive symptoms [41], it would seem important to test the level of this hormone before starting pharmacotherapy for depression. A study using another animal model of depression known as conditioned stress showed that fluoxetine, which is a selective serotonin reuptake inhibitor, reversed stress- and anxiety-related effects but did not reduce high PRL levels induced by stress [42]. This seems to confirm that the dopaminergic system plays an important role in stress-related processes. The aspect of postpartum depression, which is also associated with high PRL levels resulting from lactation, is also important. Postpartum depression (PPD) affects approximately 10% of women [43] and is closely related to endogenous hormonal secretion, and although it has been postulated to be related to estrogen, thyroxine, or autoimmune thyroid dysfunction, its strongest correlation is with high PRL and low progesterone levels [44]. It has been shown that in postpartum depression patients, increased regional grey mater volumes in the right anterior insula were positively correlated with PRL levels. Moreover, serum PRL levels served as a mediator in the relationship between the volume and symptoms of PPD [45]. Reports have linked insular functions with depressive symptoms. It was observed that the severity of the symptoms of psychomotor anhedonia, characterized by reduced satisfaction and loss of interest, was correlated with the metabolism of the right anterior insula [46]. In addition, women with postpartum depression have been shown to have impaired connectivity between the amygdala and insula when viewing pictures of their own babies, and this was accompanied by an increase in depressive symptoms [47]. Thus, it seems that hyperprolactinemia is also important in postpartum depression.

### 4.2. Schizophrenia

The most common cause of impaired PRL secretion in schizophrenia patients is antipsychotic treatment. Neuroleptics have a D2R blocking effect and can therefore increase the secretion of PRL, and drug-induced hyperprolactinemia after antipsychotic treatment is well-documented. Although an increase in PRL has also been demonstrated in untreated psychiatric patients [48,49,50,51,52,53], it is related to stress, and it might result from the increased dopamine neurotransmissions that occur during psychotic episodes [54,55]. However, the exact mechanisms by which PRL increases, even in antipsychotic-naïve patients, are not clear [56]. A correlation has also been shown between reduced PRL levels and suicide attempts in schizophrenic patients. The authors suggested that this was related to positive symptoms, particularly “paranoid” symptoms, which is consistent with the well-documented dopamine hypothesis of schizophrenia [57]. Nevertheless, it should be kept in mind that the dopaminergic hypothesis of schizophrenia relates to the mesolimbic pathway and not to the tuberoinfundibular dopaminergic pathway [58]. In a comprehensive meta-analysis, which compared thirty-two oral antipsychotics used in the acute treatment of adults with multi-episode schizophrenia, it was found that olanzapine, asenapine lurasidone, sertindole, haloperidol, amisulpride, risperidone, and paliperidone were associated with significantly increased PRL levels [59]. In a recent meta-analysis, newer antipsychotics (e.g., risperidone, amisulpride, and paliperidone) and older antipsychotics (e.g., chlorpromazine, haloperidol, and sulpiride) were shown to increase PRL levels, with a large effect size. In addition, women were more likely to have increased PRL levels after antipsychotic treatment [60]. It is not easy to down-regulate increased PRL levels in patients with schizophrenia. Usually, hyperprolactinemia is treated with oral medications with dopamine agonists [61]. Alternatively, vitamin B6 is often recommended, which has been reported to lower prolactin levels by influencing levels of dopamine. Additionally, some natural treatments that lower prolactin levels are often recommended, such as reducing alcohol intake, reducing stress with yoga or meditation, or taking herbal medicine called chaste berry (Vitex agnus-castus) [62].

## 5. Novel Aspects of PRL Action in CNS

### 5.1. Neurogenesis

The potential molecular mechanisms of prolactin action in neurogenesis and its effects on brain development have been comprehensively described elsewhere [63,64]. In the present review, we highlight some evidence indicating the direct role of prolactin in neurogenesis in the hippocampus and in the SVZ, which are believed to be crucial in adult neurogenesis. 

The occurrence of high PRL levels in the ChP should be associated with neurogenesis processes. In fact, PRL has many important functions as a neuropeptide, and it appears to be particularly important in regulating neurogenesis; however, results from studies on the effects of PRL on neurogenesis are not consistent. 

It has been shown that PRL in pregnant and postpartum female rats can enhance SVZ cell proliferation, while a disrupted PRL release inhibits SVZ neurogenesis in vivo [65]. Plasma glucocorticoid concentrations are elevated during stress and the perinatal period [66,67,68]. It appears that the observed activation of PRL under these conditions may be an important factor that counteracts the deleterious effects of glucocorticosteroids on hippocampal neurogenesis through mechanisms mediated by PRL receptors. The injection of PRL into the lateral ventricle of male mice has also been shown to increase neuronal proliferation in the dentate gyrus of the hippocampus. In addition, the exogenous administration of PRL can activate a pool of latent precursor cells in the adult mouse hippocampus [69], and studies using PRL-deficient mice have shown a reduced number of neuronal precursor cells in the hippocampus. It was also observed that the reduction in hippocampal neurogenesis induced by chronic stress was prevented by the daily administration of PRL to male mice [70]. In contrast, injections of PRL into rats for an additional 14 days after birth reduced the neurogenesis of the dentate gyrus and olfactory bulb, leading to worsening depressive states [71]. Thus, the role of PRL on neurogenesis appears to be age-dependent and requires further studies to be fully elucidated. 

### 5.2. Blood Brain Barrier and PRL

Of interest are studies on the relationship between brain barrier permeability and neuropsychiatric disorders. There are three principal barrier sites between blood and the brain. The blood–brain barrier (BBB) is a physical and biochemical barrier between blood vessels and brain tissue which protects the nervous system against unnecessary factors and enables the selective transport of substances from blood to cerebrospinal fluid. The BBB is formed by microvascular endothelial cells that line the cerebral capillaries which penetrate the brain and spinal cord. The second barrier, which protects the brain, is the blood–cerebrospinal fluid barrier (BCSFB) which is formed by epithelial cells of the ChP. Although there are several similar properties between the BBB and the BCSFB, it is important to note that the cellular basis of these two structures and their basic functions differ. The BBB is located in the capillaries of the brain and is, therefore, an endothelial structure whose main role is to protect the brain from physiological fluctuations in the plasma of various solutes, while the BCSFB is formed by a layer of modified cuboidal epithelium which secretes CSF, and this process is the main function of this epithelium. The third barrier is the arachnoid membrane. The arachnoid is avascular but lies close to the superior sagittal sinus and is separated from it by the dura mater. The cells of all barriers are connected in a complex at a junctional complex by the tight junction (TJ) and the adherens junctions (AJ); however, transport across the arachnoid membrane is not an important route for the entry of solutes into the brain [72,73]. 

Several reports link brain barrier permeability to depression or schizophrenia and susceptibility to treatment. Numerous animal models of depression and post-mortem tissues have shown that depression results from the BBB’s unsealing, as observed by decreases in TJ protein expression [74,75]. 

Schizophrenia patients also show leakage of the BBB, as observed by a decreased expression of brain barrier-related proteins (e.g., claudin-5) [76]. Moreover, positive effects of estrogen therapy have been shown in women with schizophrenia [77], which may be directly related to the effect of estrogen on BBB permeability. Estrogen regulates important pathophysiological pathways in schizophrenia, including dopamine activity, mitochondrial function, and the stress system. Estrogen deficiency has been observed in patients with schizophrenia of both sexes and is associated with increased psychotic symptoms. Moreover, secondary hyperprolactinemia induced by the use of antipsychotic drugs causes secondary estrogen deficiency.

PRL directly and actively regulates BBB and BCSF barriers. In Wistar rats whose PRL secretion was blocked by bromocriptine administration, decreases in claudin-5 and occludin protein expression were observed, which resulted in increased BBB permeability [78]. Thus, elevated PRL levels cause the BBB to become less permeable. There is a hypothesis that the lack of response to antidepressant treatment in animals with high PRL levels (in a CMS model) may be responsible for the lack of adequate permeability at the BBB. Excessive tightness of the BBB caused by a high level of PRL, among other things, may result in a reduced penetration of drugs into the brain. To date, the focus has been on blocking P-glycoprotein to increase the penetration of many drugs in the brain [79]; nevertheless, the manipulation of PRL levels may be equally important in the treatment of drug-resistant depression. Since the BCSF is formed by epithelial cells of the ChP, it would appear that PRL can be involved in regulating BCSF permeability [77]. Studies using ^125^I PRL infusion have shown high binding in the ChP, while other brain vessels were free of radioactive PRL [9]. This study confirmed, once again, that circulating PRL is transported to the brain by the BCSF. 

## 6. PRL and Other Disorders

Imbalances in the secretion of PRL can cause many diseases other than neuropsychiatric disorders. One of them is hyperprolactinemia, which is usually defined as an increased level of PRL. In clinical practice, the term hyperprolactinemia is typically used when PRL levels chronically increase. Values of >20–25 ng per ml (420–500 mIU per L) are considered pathological, but the threshold value depends on the type of test used. This disease is one of the most common endocrine dysfunctions of the HPA axis, and clinical symptoms of hyperprolactinemia include increased risks of anxiety and depression. However, increasing the level of this neurohormone may lead to recurrent headaches, mood disorders, irritability, and depressive states in women’s menstrual disorders, or secondary amenorrhea, breast pain, obesity, and skin problems. Men with hyperprolactinemia may present with hypogonadism, infertility, and hyposexuality (Table 1).

### 6.1. Hyperprolactinemia, Sexual Health, and Infertility

Hyperprolactinemia inhibits the secretion of the gonadotrophin-releasing hormone, resulting in the reduced release of follicle-stimulating, luteinizing, and testosterone hormones. Consequences include the arrest of spermatogenesis, impaired sperm motility, and a change in sperm quality [80,81,82,83]. It appears that PRL influences fertility by influencing gonadotropin-releasing hormone (GnRH) neurons [84,85]. The infusion of PRL subcutaneously in female mice resulted in hyperprolactinemia (with a mean infusion of 260 ng/mL) which was then associated with a loss of estrous cyclicity, anovulation (as reflected by a decrease in ovarian corpora lutea), a reduction in circulating luteinizing hormone (LH) and follicle-stimulating hormone (FSH) levels, and a decrease in pituitary luteinizing hormone (Lhb) and follicle-stimulating hormone (Fshb) mRNA [86].

Sustained high levels of PRL can lead to fertility problems, reduced libido, and amenorrhea because PRL affects reproductive organs. Increased prolactin levels in the blood decrease the secretion of gonadotropins, and the consequences of high PRL levels are gender-specific. In women, ovulation disorders are often encountered, including the complete disappearance of ovulation. Men suffer from erectile dysfunction, a reduced libido, and sometimes gynecomastia, i.e., the appearance of breasts. In both men and women, milk flow (or galactorrhea) from the nipple is possible. 

### 6.2. PRL and Carcinogenesis

PRL also plays a major role in breast cancer. PRL in the mammary gland stimulates tissue profiling, development, and maturation [87], and increased PRLR expression has been observed in human breast cancer cells [88,89]. Population-based studies in post-menopausal women have also shown a positive correlation between plasma PRL levels and breast cancer risk [90]. It has been reported that breast cancer is more common in female patients with schizophrenia than in the general population. Recently, an analysis of the association between cumulative exposure to prolactin-increasing drugs and breast cancer was published [91]. The obtained results revealed that long-term exposure to PRL-increasing antipsychotics, but not to PRL-sparing antipsychotics, was significantly associated with increased odds of breast cancer. Therefore, it has been postulated that monitoring prolactinemia is of great importance in women with schizophrenia who are treated with PRL-increasing antipsychotics.

Results regarding the involvement of PRL in prostate cancer are inconclusive; nevertheless, it has been shown that PRL plays a key role in the early carcinogenesis of the gland, while it does not have a significant role in the later stages of development. In addition, studies using a gremlin knockout of PRL or its receptor have not shown that this hormone plays a key role in prostate physiology in mice [92,93]. Nevertheless, there are also reports on human prostate cell lines and samples indicating the involvement of the canonical PRLR-Jak2-Stat5a/b pathway in prostate cancer tumorigenesis and progression. Increased PRL expression in the prostate, rather than in plasma, appears to be the key. It has also been shown that PRLR overexpression in the prostate of mice caused the expansion of the basal/stem cell compartment, which has been proposed to house putative prostate tumor-initiating cells, to become disorganized [94]. 

It should also be noted that about 25–40% of all pituitary adenomas are prolactinomas. This disease is quite rare; however, its main symptom is hyperprolactinemia caused by a reduced amount of dopamine that stems from compression of the stalk. The treatment of patients with pituitary tumors is mainly through dopamine agonists which normalize PRL levels, restore the function of the gonadal axis, stop galactorrhea, and significantly decrease tumor size in most patients [95].

### 6.3. PRL and Artherosclerotic Vascular Disease

Atherosclerotic vascular disease is the consequence of a chronic inflammatory process, and PRL is a component of the inflammatory response. It has been shown that hyperprolactinemia may contribute to the atherogenic phenotype [96]. This may be related to the fact that increased levels of PRL impair the function of the endothelium and reduce insulin sensitivity. Furthermore, it has been shown that high PRL levels are correlated with high arterial blood pressure, which is a recognized risk factor for cardiovascular disease [97,98]. It is interesting to note that in studies involving women with varying levels of PRL who were treated with statins (Atorvastatin), the effects of the drug depended on baseline PRL levels [99]. 

### 6.4. PRL and Autoimmune Diseases

The role of PRL has been described in many autoimmune diseases; however, few controlled analyses are available. These diseases include systemic lupus erythematosus (SLE), rheumatoid arthritis (RA), reactive arthritis, Sjögren’s syndrome, systemic sclerosis, psoriasis, Behcet’s disease, and polymyositis [100,101]. Studies on dopamine agonist treatment in humans with autoimmune diseases have been conducted only in patients with systemic lupus erythematosus, and they indicate the potential efficacy of such drugs, even during pregnancy and the postpartum period [102]. Studies on the elucidation of the mechanism of PRL action on the immune response have indicated that this hormone increases IL-2 expression on the CD4+ and CD8+ lymphocytes and thymocytes [103]. Additionally, it has been shown that PRL induces the proliferation of T lymphocytes and natural killer (NK) cells [104]. “Two faces” of PRL have also been shown in this aspect since it stimulates NK cell activity at physiological concentrations, whereas at higher concentrations, it inhibits NK cells [105].

### 6.5. PRL and Virus Infection

There are interesting studies concerning the involvement of PRL in human immunodeficiency virus (HIV). HIV is associated with hyperprolactinemia [106,107,108,109], which occurs in 21.4% of men with HIV, and it is also associated with higher CD4+ counts [106]. It has been suggested that high PRL levels may potentially cause hypogonadism in patients with HIV through the inhibitory effect of PRL on the release of the gonadotropin-releasing factor from the hypothalamus [107]. In contrast, a report indicated that hyperprolactinemia-induced hypogonadism in patients with HIV was not significantly associated with the inhibition of gonadotropin release [107]. 

Since a symptom of hyperprolactinemia is depression, there have been suggestions that depression in patients with HIV could be correlated with high PRL levels, but a recent meta-analysis disproved this [108]. 

Other studies on PRL and virus infection concern chronic hepatitis C (HCV) infection [110,111]. Studies (both in vitro and in vivo) have shown that PRL regulatory element-binding (PREB) could be regarded as a novel cofactor for HCV infection. PREB is induced by HCV and promotes the replication of HCV RNA through the formation of the HCV replication compartment [111]. This is an important discovery and provides a possible molecular mechanism of action of PRL in HCV infection. 

The recent pandemic challenge of severe acute respiratory distress syndrome coronavirus type 2 (SARS-CoV-2) induced a plethora of studies concerning the mechanisms of this viral infection, with the role of PRL in the infection mechanisms among them [112]. A study of men with COVID-19 reported high PRL and luteinizing hormone levels and low testosterone and follicle-stimulating hormone levels, which indicated primary testicular damage during active disease [113]. Besides various hypotheses concerning the mechanism responsible for high levels of PRL in COVID-19 patients, it is postulated that stress accompanying COVID-19 may induce hyperprolactinemia [112].

## 7. Conclusions

This is relatively short literature survey, and each sub-chapter deserves a review of its own. However, the data provided will make readers more aware of the role of PRL in various stress-related disorders. As the data described above indicate, PRL impacts many physiological processes and plays a role in various diseases, although its mechanisms of action are still the subject of research. Nevertheless, stabilizing PRL levels, which are dysregulated under different pathological conditions, has significant therapeutic potential. In addition, the endogenous level of PRL may serve as a predictor of a patient’s response to pharmacotherapy, which was shown in the case of depression. 

## Figures and Tables

**Table 1:** The symptoms of hyperprolactinemia distinguished by gender.

Gender	Symptoms
**Women**	Menstrual cycle disorders—irregular menstruation, amenorrhea, and anovulatory cycles Galactorrhea—milk production in women who are not pregnant or breastfeeding Hypogonadism—hormonal failure of the ovaries Sexual dysfunction—decreased libido, disturbances in the agitation phase, problems with lubrication and pain during sexual intercourse, and orgasm disorders Fibrocystic changes in the breast Mastalgia—breast pain Outbreak symptoms, e.g., hot flushes Bone decalcification
**Men**	Sexual disorders—decreased libido Erectile dysfunction Gynecomastia—enlargement of one or both breasts, rarely with galactorrhea Reduction in muscle mass and hair loss in the genital area
**Symptoms occurring in both sexes**	
	Weight gain Infertility Osteopenia, i.e., a decrease in bone mineral density Headaches Visual disturbances Obesity Deposition of fatty tissue in the abdomen Anxiety Depression

## Data Availability

No new data were created or analyzed in this study. Data sharing is not applicable to this article.
